# A Few Brief Observations on the Pathology of the Triple Articulations of the Shoulder

**Published:** 1898-10

**Authors:** Thomas H. Manley

**Affiliations:** New York


### A FEW BRIEF OBSERVATIONS ON THE PATHOLOGY OF THE TRIPLE ARTICULATIONS OF THE SHOULDER.

By THOMAS H. MANLEY, M.D.,

New York.

The morbid changes succeeding the application of violence, or coincident with diseased conditions of the shoulder, have always been a subject of great interest, especially since Charcot, Duchenne and others have demonstrated that when the heads of the bones or the articulations are involved the etiological factors in operation may be of central origin. But it will be my purpose in the present instance to very briefly review a few only of the more salient features noted in connection with the ordinary lesions centered in the alar appendages or the shoulder, after injuries.

*Stricture.*—The shoulder is made up of the bones for a framework, the clavicle, scapula and humerus. *The collar-bone* is a horizontal, lateral girder, which serves the purpose only of imparting squareness and contour to the shoulder. It is necessary neither for agility nor strength; in fact, it is an impediment to both, as the fleetest, most powerful, or climbing quadrupeds are without it. *The scapula,* or shoulder-blade, is an osseous plate with a series of surfaces, borders and spurs of bone. It serves as a base of support and fulcrum to the humerus. *The humerus* has *two* heads, an *intra*-capsular and *extra*-capsular, with *two* necks, an anatomical and surgical.

The shoulder has three articulations, all anatomically atypical. First, the costo-scapular, capable of permitting a wide range of motion; second, the scapulo-clavicular, a double-joint of very limited motion; third, the humero-scapular, quite identical with the costo-scapular articulation, in being devoid of ligaments and solely supported by muscles, although this has a large, strong capsule. It is therefore absurd and misleading to speak of "the shoulder-joint," for there is no such structure, but rather "shoulder-*joints.*"

*Nerves.*—The powerful nerve-cords coming down to supply the upper extremity have a large proportion of fibrous tissue in their composition, and hence tend to serve as ligaments in holding the shoulder up anteriorly. They pass down under the clavicle and in the axillary-space lie in loose myxomatous tissues, at such a distance from its articulation as to in no manner impede joint action. Next to the muscles the nerves here, are most exposed to injury in the application of violence.

*Vascular Supply.*—The arterial supply of the shoulder is quite entirely from the subclavian and axillary. The large blood-vessels in the axillary-space are noticed to pursue a somewhat winding course and are in close contact with the larger nerve-cords. These vessels in the axillary-space are freely movable and take such a course as to escape over-tension, compression or laceration in many of the severe forms of shoulder traumatisms. The lower two-thirds of the axillary vein, in its inner and inferior aspects, is in close contact with the lymph-ganglia. Quite the entire arterial supply to the forearm passes down through the axillary-space, but the venous return pours upward through two different channels—the median cephalic passing up over the deltoid to discharge its contents into the first part of the axillary in the center of the costo-coracoid membrane, the basilic coursing up the arm to join the brachial vena-comes and form the axillary vein.

*The Muscular Structures.*—The muscles playing in the shoulder are principally of two kinds: one, large and powerful, employed in support and leverage, as the trapezius, the deltoid and pectoralis major; the other of minor development, which rather serves the purpose of ligaments, as the subclavius, the lesser pectorals, and the internal rotators. The trapezius and deltoid, which serve powerfully retentive as well as contractile purposes, have a large admixture of aponeurotic structure. Besides the preceding structures, bursæ or larger accessory synovial pouches and lymphatic organs deserve special consideration, the former in consequence of their tendency to inflammatory changes after trauma, and the latter because of their invasion in specific diseases.

The preceding very brief and incomplete sketch of the anatomical structures is necessary before it is possible to deal with morbid changes. Did space permit, much might be said on the complex functions or physiology of the parts.

With a structure so exposed as the shoulder, of its remarkable strength, its complex mechanism and great activity, we may expect to find it the frequent seat of many pathological changes after the application of violence.

*Results of Violence to the Shoulder.*—First, *sprains.* Sprains of the shoulder are as diverse in their varieties here as are the different types of fracture. They differ not only in degree, but in the structures involved, and hence in some their full force falls on the articulations, in others on the periarthritic structures; at one time the muscles and another the nerves, the bursa or bones, much depending on the quality and volume of force—on whether there has been a contusion, torsion or deep laceration. In severe forms the bones are fissured, the smaller blood-vessels opened, nerves torn, the muscles lacerated or torn from their attachments.

The pathological changes succeeding are *inflammatory* or *non-inflammatory.* The latter are always of more serious import, as they point to extensive nerve lesion.

The acute changes attendant on inflammatory action involve chiefly the connective tissues and are most accentuated when the bursæ have suffered violence. The parts over the joints are ecchymosed, tumefied and sensitive. Muscular rigidity is marked and joint action is limited.

In severe cases there has been an effusion of blood into the muscle-spaces and the osseous parts have shared in the damage. Of late, since the Roentgen rays have been employed to clarify diagnosis, oftentimes what had been regarded as simple sprains have been found associated with non-displaced fracture. (Bilhaut, *Jourde*, Dr. *Orthopœdique*, javice 1897.)

In certain cases the extent of tumefaction immediately following is so great as to render definite examination impossible without an anesthetic. In hysterical, neuralgic or rheumatic subjects the degree of pain borne is great and its course is very protracted.

Non-inflammatory types of sprain are generally those of a serious character at the outset. In these we have a history rather of a twist or violent over-tension of the parts than a bruise from a blow or fall. All the soft parts have been evidently overstretched; there has been a condition of arthritic ectasis.

After this type of sprain the most pronounced and palpable signs of pathological changes are, first, *muscular atrophy*, a marked, visible wasting, or an interstitial softening sensible to the touch. With the body in the upright attitude, the levator muscles yield and there is a marked drooping of the whole shoulder, which, however, is more or less fixed.

*Muscular rigidity* is at once noted when an attempt is made to begin full joint action. This condition is somewhat overcome by pulmonary anesthesia; in some, however, only slightly. It may remain over a long period. Until it disappears, restoration of function is impossible.

The most prolific cause of this condition is dependent on muscular changes, either interstitial or peripheral, degeneration or absorption of the muscular fibers, with a contracted, shortened state of the entire muscle.

One of the most frequent causes of muscular rigidity is intermuscular adhesions; the inflammatory deposits, having undergone condensation, glue the muscle sheaths together, so that their free-gliding action on each other is impeded. These adhesions seriously embarrass the free movement of the circulatory current, and thus indirectly impair the nutrition of the affected limb. It may be well to note, in passing, that a similar state of the limb may ensue after various erratic types of muscular rheumatism, especially in children, and be mistaken for genuine joint disease.

*Psychical symptoms* are quite invariably present in those cases which pursue a chronic course. The afflicted are despondent and are prone to believe that their cases are incurable, that they will become dependents, and that the impaired limb may remain feeble; hence the impression on the system is marked, their digestion fails, and anemia is pronounced.

*Dislocations, Complete and Incomplete.*—A dislocation is a *sprain* plus a displacement of bone.

At the shoulder we have *two* typical dislocations, one of the humerus and the other of the clavicle.

The humeral luxations are quite entirely dependent on muscular damage, the clavicular on a rending of the ligaments.

In humero-scapula dislocations it is of fundamental importance to remember that they are *complete* and *incomplete*, the latter by all odds the most common and most manageable. In these but one muscle is seriously compromised. The deltoid is overstretched or paralyzed, the capsule is stretched but not torn, some part of the articular surface of the inner humeral head is yet in contact with the glenoid surface. The massive deltoid *alone* holds the humerus well up under the acromion vault. This any one can readily demonstrate on the cadaver. Let this muscle be completely divided by a transverse incision, with its base deflected upward, when at once the humeral head leaves the glenoid cavity, passing down over the root of the tricepital head into the axilla, or forward out under the coronoid spur.

By this simple procedure, too, the lax distensile property of the capsule is readily demonstrated. The tenacious, long head of the biceps offers some resistance to forward advance, but it is a yielding structure, which rather steadies the humeral head than exerts a positive tension over it.

In complete luxations the extent of damage to the deep structures is often, no doubt, very considerable; the deep rotators are torn from their attachments, important nerve trunks are lacerated, and the capsule suffers at times great damage. Cases are on record in which the capsule has been completely torn from the anatomical neck, widely opened or rent in two.

But, that it is always opened or torn off in complete dislocations is certainly not the case. This was demonstrated last summer in a case brought to me by Dr. W. G. Gaudineer, of this city. The patient was a child of six years. He had a complete luxation of the humerus for three months. The head made extensive excursions forward under the clavicle and down into the axilla, completely out of the cavity; yet on dissection the capsule was found entirely intact.

In these dislocations, besides the great damage to the muscles and nerve branches, the three great nerve-cords may sustain grave or irreparable injury. The brachial plexus may be violently overstretched, contused or ruptured. But, as nerve-tissue possesses a most remarkable resistance to trauma and its recuperative property is marvelous, we are not able to precisely demonstrate at the time of injury, or later, just how far this participation of nerve injury proceeds.

Striped muscular tissue is not prone to inflammation, but when it develops in consequence of physical violence it pursues a chronic course. Therefore, as the muscles almost alone bear the brunt of all types of humero-scapula luxations, we may expect to find any degree and variety of pathologic changes in their substance. For weeks and months, sometimes indefinitely, after injury they remain weak, painful and wasted, and hence the joint never quite fully recovers its function. With the deltoid ruptured, permanent, complete reduction of the humeral head is impossible. The counteracting muscles, preserving their integrity, keep up a pull with nothing to resist them. In vain violent and repeated efforts at reduction are made; but instantly on releasing force the glenoid hollow is found empty. Sometimes, under these circumstances, it is said that the humeral head has "button-holed" through the capsule, but I have been unable to gather from the recorded cases of arthrotomy for dislocation of the humerus any such an anatomical condition. It has been my privilege to witness five arthrotomies by various surgeons in America and Europe for irreducible humero-scapula dislocations, but in none was there any button-holing of the capsule.

The pathological conditions consecutive to humero-scapula dislocations are manifold and depend upon two factors, the results of the trauma and constitutional states. From trauma may result arthritis, periarthritic inflammation, ostitis and periostitis, muscular atrophy, paralysis and vascular changes.

*Constitutional Influence as a Complicating Factor.*—In all severe joint injuries, whether attended with luxations, fractures or not, the constitutional state plays an important *rôle;* constitutional or acquired tendencies and the various cachexias may leave their impress here.

In some instances it is most extraordinary to note the wide-spread constitutional disturbances which may succeed these traumatisms; the profound impression on the sensorium and on the whole spinal system of nerves, the extreme degree of inflammation provoked, the marked rigidity, anchylosis or extensive atrophic wasting of the limb.

That vague, protean, obscure state of the system known as hysteria here, exercises a most potent influence. Rheumatism comes next. In every instance presenting intense reaction, running into a chronic state, it is well not to overlook the possibility of coincident gonorrhea, the tubercular diathesis or specific taint.

*The Disorganization of Structure.*—Bearing in mind that there are clinically *two* types of humero-scapula dislocations, the *complete* and the *incomplete*—and this is essentially peculiar to dislocations at this articulation—we may expect a very wide difference in the degree and character of physical disorganization sustained.

In incomplete dislocations here the primary, dominant pathological feature present is an over-tension of the deltoid muscle, with probable rupture of many fasciculi in its vertical or acromion segment. The capsule is yet completely intact, and the articular head has not moved entirely out of its socket. The great nerve-cords have been overstretched, but none of their important pouches have been torn off. The bursæ have all suffered compression, and the periarthritic structures have sustained contusion.

The consecutive changes in this subluxation are first *inflammatory* and secondly *atrophic.*

The inflamed, overstrained deltoid has become relaxed and allows the head of the humerus to roll forward or downward, out of the hollow of the glenoid cavity. This tendency to displacement is augmented by a free effusion of sero-sanguinous fluid into the synovial sac.

With a proper appreciation of these conditions, it is readily apparent why our efforts at complete restoration are futile and harmful, when the state of the surface points to a large synovial effusion of either a sanguinous or serous substance. Superadded to this is a multiple neuritis and trophic changes involving, in various degrees, the muscular components of this joint.

In *complete* humero-scapula luxations, superadded to the effects of sprain and to the violent over-tension of subluxation, are the extensive laceration of parts vital to the integrity of the joint.

It is true, as was demonstrated in a case of my own last summer, that we may have extensive excursions of the head of the humerus, far away from the glenoid cavity, without the capsule giving way; but, from the few cases on record permitting of dissection early after the accident, it is clearly demonstrated that in most instances of complete dislocation the capsule is opened and the head passes through it, or the capsule is torn off, partly or completely, either at the anatomical neck or at the border of the glenoid fossa. In this event, when the humeral head is driven far forward across the coracoid arch, the long head of the biceps is torn off, some part of the tendon of the subscapularis has sundered, and it is impossible that the musculo-spiral and circumflex nerves can escape serious damage. I can find no case on record where the blood trunks have been seriously compromised by a dislocation here, though the number is large of the recorded cases in which they have suffered various degrees of disorganization and destruction in *violent* efforts at reduction.

*Dislocations at the scapulo-clavicular joint* involve no important structures except the ligaments, and hence the pathological state resulting is devoid of those features dominant in other dislocations. Although the acromion end of the clavicle is restricted in its upward range of action, it may be said that its fixed position depends quite exclusively on ligaments. When the acromion head of the clavicle is completely displaced its duplex capsule is quite completely disorganized, and therefore the rotary movement of the shoulder on the central plane of the body is greatly diminished, if not entirely lost.

This type of dislocation derives its greatest interest from the great tendency to overlook it, especially in fat subjects, on a superficial examination; and besides, because when of an aggravated character it cannot be so treated as to effect complete and permanent reduction.

Dislocation or displacement of the shoulder *en masse* is never seen, though partial displacements are not infrequent. They are of three kinds: First, and most common, those seen after any severe injury of the shoulder, when it droops downward and sinks forward and inward; second, when the lower angle of the scapula projects outward from a displacement of the latissimus-dorsi muscles; third, after operations, those for mammary cancers, which entail the excision of the pectoral muscles and costo-coracoid fascia, when the equipoise of the shoulder is lost and it is drawn backward by the serratus-magnus, rhomboid and levator of the scapula muscles.

When, from any cause, the nerve-supply of the parts are involved by disease, in rheumatic or other process of inflammation seizing on and inducing trophic changes in the muscles, marked deviations in the position of the shoulder are noticed.

SUMMARY.

Sprains, twists, contusions and dislocations at the shoulder constitute the most important class of traumatisms that we are called upon to treat in the upper extremity.

In aggravated form the whole system suffers violent disturbance, strength and functions are impaired, and permanent organic changes may succeed.

In consequence of the mechanical construction of the shoulder and its triple-jointed arrangement, limitation in range of action in any one articulation is, in varying degrees, compensated for, and hence but moderate deformity or physical impediment permanently results.

The shoulder being a structure in close relation to the center of circulation, with an abundant vascular supply, in the absence of pathological conditions of a constitutional origin, is generally susceptible to prompt reparative processes.

Being an appendage of the body in an exposed, independent, suspended position, it is susceptible to a rigorous examination and the application of direct and energetic treatment.

The principal pathological conditions calling for remedial aids after the injuries enumerated depend on, first, and the preponderance of cases, damage to the muscles, contusion, over-tension or laceration; second, neural, or injury to the nerves, the motor, sensory, or trophic; third, the arthritic structures; fourth, the osseous structures, the periosteum under the attachment of the muscles or tendons, or the vascular cancellus structures which constitute the articular surfaces; fifth, injury of the bursæ, with or without a propagation of inflammatory changes into the articulations; sixth, a traumatic arthritis—a necessary part of every dislocation—the coincident association of psychical manifestations is important to note under many circumstances; seventh, in order that treatment may be effective and a safe forecast of results may be made, it is imperative that the existence or tendency to various constitutional diseases be sought for.

*115 West 49th St.*

